# Reimagining and reinvesting in rural hospital markets

**DOI:** 10.1111/1475-6773.14047

**Published:** 2022-08-21

**Authors:** Caitlin Carroll, Arrianna Planey, Katy B. Kozhimannil

**Affiliations:** ^1^ Division of Health Policy and Management University of Minnesota School of Public Health Minneapolis Minnesota USA; ^2^ Department of Health Policy and Management UNC Gillings School of Global Public Health Chapel Hill North Carolina USA

## 1 INTRODUCTION

A key issue facing the United States is how to structure rural health care delivery. In research and policy discussions of this challenge, a core focus has been the appropriate role for rural hospitals. In rural areas, hospitals face structural barriers to delivering care: low patient volumes limit revenues and drive up the cost of care delivery, leading to closure and undermining surviving hospitals' capacity to provide high‐quality care.^1^ Yet, losing access to hospital care can be detrimental to the health of local residents.^2^, ^3^, ^4^ Rural hospitals also provide important services in times of crisis, most recently during the COVID‐19 pandemic, when there is an influx of patient needs.^5^, ^6^, ^7^

These issues raise two core questions about rural health care. First, what hospital infrastructure is needed in rural areas? While decreased access to care can lead to treatment delay, not all rural hospitals can safely provide the full suite of health care services. Some rural residents may benefit from a health care system that facilitates referral to care outside the community, limiting local care delivery. Second, how should rural hospital care be financed? Low patient volumes make rural hospital services inherently expensive, given the high fixed costs of care provision. Considering when increased financing is warranted and what form additional financing should take is also an essential component of policy discussion.

In this commentary, we discuss major issues facing rural hospitals and outline possible roles for public policy in restructuring rural hospital care. While hospital services are an important component of rural health care, there are many determinants of rural health. As rural hospitals are considered in research and policy debates, attention should expand beyond hospital services to imagine how the broader health care and public health infrastructure can support rural health.

## 2 ACCESS TO CARE: HOSPITAL AND SERVICE LINE CLOSURES

A core challenge in rural communities is declining access to hospital care. Eight percent of rural hospitals have closed over the last decade, and closure rates have increased in recent years.^8^ Even hospitals that remain open are downsizing and closing individual service lines. Only 40% of rural counties had obstetric services in 2018, for example, down from 55% in 2004 and 75% in the 1980s.^9^, ^10^ Among rural communities, reductions in access have not been not equally distributed.^11^ Since 2010, rural counties with hospital closures have had above‐median shares of racialized residents, including Black and Hispanic people.^12^ Similarly, obstetric unit closures have disproportionately affected rural communities with a higher proportion of Black residents, those in more remote areas, and in states with less generous state Medicaid programs.^13^, ^14^

Promoting access to care in areas with low population density is inherently challenging. Even with recent closure trends, rural hospitals have more unoccupied beds than urban hospitals, suggesting little strain on aggregate availability.^15^ However, travel distances to access hospital care in rural areas can be substantial. Thus, a challenge in rural areas is that hospital beds may be oversupplied at the market level but undersupplied in local communities that face heavy travel burdens. Importantly, access to hospital beds does not necessarily guarantee access to adequate care. Work by Hegland et al. in this issue shows that rural hospital markets have more bed capacity per capita and lower occupancy rates than urban areas but less access to other hospital resources such as capital funding and staffing.^16^

Hospital closures are concerning, in part, because of the potential effects on patient health. On the one hand, hospital closure decreases access to care and increases travel times, raising concerns about adverse health outcomes for patients with time‐sensitive conditions. This may be exacerbated for Black or Latinx residents, who face additional barriers to access, such as longer travel distances and the higher likelihood of ambulance diversion.^17^, ^18^ On the other hand, if hospitals that close tend to provide low‐quality care, then closure may improve health outcomes by reallocating patients to higher quality facilities. Even if closed hospitals have similar performance as their competitors (prior to closure), the aggregation of volume at surviving facilities can improve the quality or efficiency of care in some service lines since facilities can better leverage economies of scale.^19^, ^20^

Despite sustained policy interest in rural hospital closures, evidence on its clinical effects is relatively limited. Research shows that closure can increase mortality for patients with time‐sensitive conditions such as heart attacks or childbirth, although evidence is mixed.^2^, ^3^, ^4^, ^21^ However, the implications of hospital closure for patient health outside of these clinical settings are unknown. An important question is how hospital closure impacts patients with chronic health conditions, who may be particularly affected by the loss of hospital services, as well as the reduced supply of physicians that can accompany hospital closure.^22^ Further work on the relationship between hospital closure and access to outpatient services will be important for understanding the full impact of closures.^23^

Beyond the clinical context, rural hospital closures can also affect social determinants of health, including local economies. Rural hospitals are often major employers in rural areas, so closure can affect the economic vitality of the local community. Recent empirical work has provided new evidence on this dynamic. Alexander and Richards, and in this issue, Chatterjee et al. find that health care employment decreases after hospital closure but find few effects on the economy outside of the health care sector.^24^, ^25^ Vogler and Malone et al. estimate decreases in labor force participation after hospital closure, suggesting a mechanism behind the decrease in employment.^26^, ^27^ Importantly, much of the recent work has found evidence of economic decline before hospital closure, suggesting that local economic conditions may be contributing to rural hospital strain.

## 3 EVOLUTION OF RURAL HOSPITAL MARKETS: CONSOLIDATION AND BYPASS

Beyond closures, rural hospital markets have been characterized by two important dynamics: consolidation (a supply‐side phenomenon wherein hospitals merge or change ownership) and bypass (a demand‐side phenomenon whereby rural residents do not seek care at local rural hospitals, “bypassing” them in favor of traveling to urban hospitals).^28^

Consolidation of rural hospitals into systems has increased over time. About 11% of rural hospitals merged between 2005 and 2016, and the effect of this consolidation is unclear.^29^ Systems have greater financial resources, so system affiliation can serve as an alternative to closure, improve infrastructure investment at rural hospitals, and help build referral networks.^30^ However, hospital mergers increase market power and lead to increased prices.^31^ There is also concern that systems acquire rural hospitals with the intent of downsizing or shutting them down; while strategic downsizing may address costly duplication of services, it can also drain resources from the local community. Recent work has found evidence to support both sides of this debate: rural hospital mergers are associated with improved outcomes for patients with time‐sensitive health conditions such as heart attack and stroke, increased availability of technology, and increased capital expenditures, but there is also evidence that systems eliminate services at their acquired hospitals.^32^, ^33^, ^34^, ^35^ More work is needed to understand the tradeoffs from consolidation in rural areas and implications for antitrust policy, given that competition may be inherently limited in rural markets.

Another important consideration in rural markets is rural hospital bypass. About a third of Medicare inpatient stays were not at beneficiaries' closest hospital in 2018, even when services were available at those closest hospitals.^36^ A key question is how much bypass has contributed to overall decreases in rural hospital volume. Friedman and Holmes, in this issue, argue that increases in bypass over time explain more of the overall volume decrease than declines in population or shifts toward outpatient care.^37^ They also find that rural residents are more likely to seek care at urban hospitals if their nearest hospital affiliates with a system. This pattern could reflect a distaste for system affiliation by local residents or reflect a greater regionalization of care, where system affiliation enhances connections between local rural areas and urban care centers.

Bypass has received attention in research and policy circles because it threatens rural hospital finances, given the decrease in patient volumes. There has been less focus on how bypass impacts outcomes for rural patients. For some services, the costs of additional travel may be outweighed by the benefits of receiving more specialized care in an urban area. To some extent, bypass signals a higher perceived quality of urban hospitals by rural residents.^38^ Further research on the tradeoffs of bypass for the health of rural residents is needed and can help inform debates about care regionalization in rural markets. Understanding the equity implications of the bypass would also be valuable; the costs and benefits of bypass may accrue inequitably along the lines of race, class, and insurance type since not all rural residents are able to travel for care.^39^, ^40^

## 4 QUALITY OF CARE AT RURAL HOSPITALS

A core issue is how to ensure high‐quality care at rural hospitals, given the difficulty of financing capital investments and recruiting and retaining clinical staff. With few patients, there are also limited opportunities for clinicians at rural hospitals to practice and maintain clinical skills, especially for specialized care. Lower volume facilities may not always be well‐equipped to handle higher needs patients.^41^, ^42^, ^43^

Overall, evidence on the quality of care at rural hospitals is limited and mixed. Consistent with the challenges outlined above, rural hospitals are less likely to house expensive technologies – such as intensive care units, cardiac catheterization labs, coronary artery bypass graft surgery, robotic surgery, and electronic health records.^44^, ^45^, ^46^, ^47^ There is also evidence that rural Critical Access Hospitals have worse risk‐adjusted mortality for select health conditions, relative to urban hospitals, but fewer complications after surgical procedures.^48^, ^49^ However, quality measurement at rural hospitals poses several challenges. Low patient volumes lead to noisy measurement, and risk‐adjustment may not fully control for differences in patient populations across rural and urban hospitals.^50^ For example, distance traveled to care is related to health outcomes and differs across rural and urban areas but is not included in risk adjustment. Moreover, common quality metrics are generally developed and tested at urban hospitals and may not reflect important functions at rural hospitals, such as stabilization and transfer activities.^51^, ^52^, ^53^

## 5 DISCUSSION: PUBLIC POLICY IN RURAL HOSPITAL MARKETS

A key question for policy makers is what role should hospitals play in rural health care delivery. The first consideration is what types of hospital services should be maintained locally and what types of services should be provided regionally.^54^ For some services, local access is essential, for example, emergency care. For chronic or complex conditions, however, the benefit of receiving treatment at larger, regional facilities may outweigh the cost of traveling farther for care. These dynamics suggest a reimagination of rural hospitals, which might have limited local care delivery but enhanced resources for facilitating referrals to care outside of the market. Yet, there is little evidence on which service lines are valuable to maintain in local areas. Research on the effects of service disruptions is limited to select, time‐sensitive health conditions such as heart attack or childbirth.^2^, ^3^, ^4^ Moreover, there is little understanding about how to facilitate care for patients who must seek treatment outside of their local areas.

Policies that allow limited service provision at rural hospitals have advanced in recent years. The Rural Emergency Hospital Program, for example, will allow rural hospitals to eliminate their inpatient service entirely in exchange for increased payments for outpatient care beginning in 2023. This program represents a significant departure from previous rural health policies, which have largely aimed at preserving rural hospital infrastructure. In principle, this new form of a rural hospital can reduce the cost of hospital care while maintaining access to valuable local services. Understanding how this program affects costs and patient health will be important for future policy making.

A second consideration is how much financial support is warranted for rural hospitals and what form that support should take. Proponents of increased spending on rural hospitals argue that it can protect access and facilitate quality improvements at hospitals that struggle to recruit staff and invest in new technology. Others argue that maintaining rural hospital infrastructure is costly relative to the number of patients affected, given the high fixed costs and low patient volumes at rural hospitals. This latter view suggests that financial support should be scaled back to target isolated hospitals or valuable service lines or be redirected to other community or social services.^55^, ^56^

To date, policy makers have largely responded to financial challenges at rural hospitals by increasing payments. The Critical Access Hospital (CAH) program, for example, allows rural hospitals to receive cost‐based reimbursement from Medicare rather than prospective payments per admission. Half of the rural hospitals are CAHs, and the program costs approximately $1 billion per year.^57^ Despite the scale of the CAH program, there is little evidence on its effects. Existing work has found a decrease in hospital closure under the CAH program, but less is known about the effect of additional CAH revenue on the quality of care at participating hospitals.^2^, ^58^, ^59^

Recently, policy makers have considered new payment models for rural hospitals. One payment reform under consideration is low‐volume payment adjustments. This model has been considered as a strategy for promoting access to rural obstetric care but could be flexibly applied to other service lines or bed types.^60^, ^61^ A second payment reform is a two‐part financing model: Under the Rural Emergency Hospital program, for example, Medicare will pay a lump sum to the facility to cover overhead, plus a fee for each patient visit.^62^ This system moves toward infrastructure‐based payments, rather than patient‐based payments that disadvantage low‐volume providers.^1^ In addition, incentives for efficiency under the two‐part system would likely be greater than under cost‐based reimbursement, which rewards hospitals for increased expenses. Investigating how rural hospital performance responds to different payment structures will be a top priority for future research.

Payment incentives for hospital care can also be a part of broader health equity strategies in rural health care. Alternative payment models could focus investments on rural communities that have faced historical and contemporary challenges of racism, for example, rural Black farming communities in the Southeast and rural American Indian reservation communities.^12^, ^63^, ^64^ Investments in rural health care infrastructure must account for racial inequities.^54^, ^65^, ^66^ To date, “race‐neutral” rural health policies have served to uphold longstanding racial inequities.^67^ Additional research into the racial equity impacts of existing payment reforms would inform strategies for narrowing racial and ethnic disparities among rural residents.^63^

## 6 CONCLUSION

Rural hospitals provide essential services in rural communities but are struggling to respond to changing demand for health care under the current policy environment. A key question for research and policy is what role hospitals should play in rural health care delivery. While preserving access to full‐service hospitals may be appropriate in some rural communities, others would benefit from care regionalization with targeted local care delivery. Future research can help inform policies that reimagine hospital care and health care delivery to support the well‐being of rural residents.

## FUNDING INFORMATION

No funding to report.
